# A prospective study of daclatasvir and sofosbuvir in chronic HCV-infected kidney transplant recipients

**DOI:** 10.1186/s12882-019-1218-0

**Published:** 2019-02-04

**Authors:** Michael Duerr, Eva V. Schrezenmeier, Lukas J. Lehner, Léon Bergfeld, Petra Glander, Stephan R. Marticorena Garcia, Christian E. Althoff, Ingolf Sack, Susanne Brakemeier, Kai-Uwe Eckardt, Klemens Budde, Fabian Halleck

**Affiliations:** 1 Department of Nephrology and Medical Intensive Care, Charité Universitätsmedizin Berlin, Augustenburger Platz, 13353 Berlin, Germany; 2 Department of Radiology, Charité Universitätsmedizin Berlin, Berlin, Germany

**Keywords:** Kidney transplantation, HCV infection, Direct-acting antivirals, Daclatasvir, Sofosbuvir

## Abstract

**Background:**

Only a few prospective trials exist regarding the use of novel direct-acting antiviral agents (DAAs) in kidney transplant recipients (KTR) with chronic hepatitis C virus (HCV) infection.

**Methods:**

This prospective single-center trial evaluated treatment with daclatasvir (DCV) and sofosbuvir (SOF) over 12 weeks in 16 adult chronic HCV infected KTR and eGFR > 30 ml/min/1.73m^2^. Primary endpoint was sustained virological response 12 weeks after end of therapy (SVR12). Beside baseline liver biopsy, hepatic function and glucose metabolism were regularly assessed.

**Results:**

Four of 16 study patients had previously failed interferon-based HCV treatment. Liver biopsy showed mostly moderate fibrosis score before therapy with DCV/SOF was initiated at a median of 10.3 years after transplantation. In total, 15 of 16 KTR achieved SVR12. One patient showed early viral relapse because of resistance-associated variants (RAVs) in the HCV NS5A region. Rescue treatment with SOF/velpatasvir/voxilaprevir resulted in SVR12. DAAs treatment led to significant improvement of liver metabolism and glucose tolerance accompanied with no therapy-associated major adverse events and excellent tolerability.

**Conclusions:**

Our study demonstrates safety, efficacy and functional benefit of DCV/SOF treatment in KTR with chronic HCV infection. We provide data on rescue strategies for treatment failures due to present RAVs and amelioration of hepatic function and glucose tolerance.

**Trial registration:**

Registry name: European Clinical Trials Register; Trial registry number (Eudra-CT): 2014–004551-32, Registration date: Aug 28th 2015.

## Background

Chronic hepatitis C Virus (HCV) infection represents an additional disease burden for affected kidney transplant recipients (KTR) with a negative impact on patient and graft survival [1–3]. There are a variety of long-term consequences of chronic HCV infection such as liver function impairment, consecutive liver fibrosis and cirrhosis and hepatocellular carcinoma. In addition, HCV-associated extra-hepatic manifestations can lead to premature renal allograft loss, e.g. due to recurrence of HCV-associated membranoproliferative glomerulonephritis, post-transplant diabetes, a higher incidence of rejections and post-transplant malignancies [4–7]. Before the approval of the novel direct-acting antivirals (DAAs) pegylated interferon (pegIFN) and ribavirin (RBV) were used for treatment of chronic HCV infection. However, these drugs had low efficacy with frequent treatment failures, persistent HCV replication or viral relapse. In addition, multiple severe side effects caused a high rate of drug discontinuations. In particular, the immunomodulatory properties of pegIFN are associated with a higher risk of acute rejection and increased rates of graft loss [8, 9]. Thus, pegIFN was not considered suitable for KTR while RBV alone does not result in a sustained HCV clearance.

With the development of the novel DAAs, treatment efficacy improved and drug-related side effects dramatically decreased in HCV-positive, non-organ-transplanted patients [10, 11]. Daclatasvir (DCV) inhibits HCV RNA replication by specific inhibition of the viral NS5A protein. It was approved (2014 by EMA, 2015 by FDA) and is currently recommended for treatment of chronic HCV infection of genotypes 1–6 in combination with sofosbuvir (SOF), an inhibitor of the viral NS5B protein [12]. Both, NS5A and NS5B, are critical for viral transcription and translation [13].

The novel IFN-free, pan-genotypic combination regimen with DCV/SOF demonstrated robust and permanent HCV clearance also in advanced liver disease or HIV co-infected patients [14–17]. In KTR different SOF-based combination therapies already have been reported mostly in retrospective case series to cure chronic HCV infections in KTR [18–22]. However, prospective data on treatment with DCV/SOF in KTR with chronic HCV are limited.

Here we report the results of a prospective open-labeled trial to evaluate the efficacy and safety of a fixed dose 12-weeks regimen of DCV/SOF in HCV-infected KTR. Besides safety and efficacy, changes in hepatic and extra-hepatic parameters, glucose tolerance and possible drug-drug interactions are analyzed in detail.

## Methods

### Study design and treatment

In 2016 a prospective phase II, single-center, open-label trial (Eudra-CT number: 2014–004551-32) was started at our center. In total, 16 KTR with chronic HCV infection received a 12-weeks course of DCV 60 mg and SOF 400 mg orally once daily given, followed by an additional 24-week observational follow-up period. Potential trial participants agreed to participate in the study by providing written informed consent after approval by German health authorities and an independent Ethic committee (15/0446EK15; 4,040,892). The study was conducted according the Declaration of Helsinki, the International Conference on Harmonization and Good Clinical Practice guidelines.

### Inclusion criteria

We offered treatment to all adult KTR (age > 18 years) of our outpatient clinic with chronic HCV infection and stable graft function for more than 12 months, defined as eGFR > 30 ml/min/1.73m^2^ using the CKD-EPI formula [23]. KTR were either treatment naïve or had previously failed treatment with any former regimen (without the use of novel DAAs). Chronic HCV infection was defined by > 3 months of positivity for anti-HCV antibody and HCV RNA viral load.

### Exclusion criteria

KTR with any contraindications DCV/SOF, evidence for chronic liver disease other than HCV and KTR with Child-Pugh Class B or C (Score > 6) were excluded from the study. Further exclusion criteria were: severe cardiac disease, malignancies in the last 5 years, any blood transfusions within 4 weeks, recent (within 6 months) drug or alcohol abuse (defined by [24]), coinfection with HIV or HBV, severe rejection (≥Banff II) or recurrent acute or chronic rejection within 6 months, neutrophils ≤1500/mL, platelets ≤75.000/mL, ALT >5x upper limit of normal (ULN), direct bilirubin >3xULN, Albumin < 3.0 g/dL.

### Efficacy endpoints

The primary outcome was sustained virological response at week 12 after end of treatment (SVR12). SVR was defined as undetectable HCV RNA in a study participant with previously quantifiable or detectable HCV RNA.

**Fig. 1:**
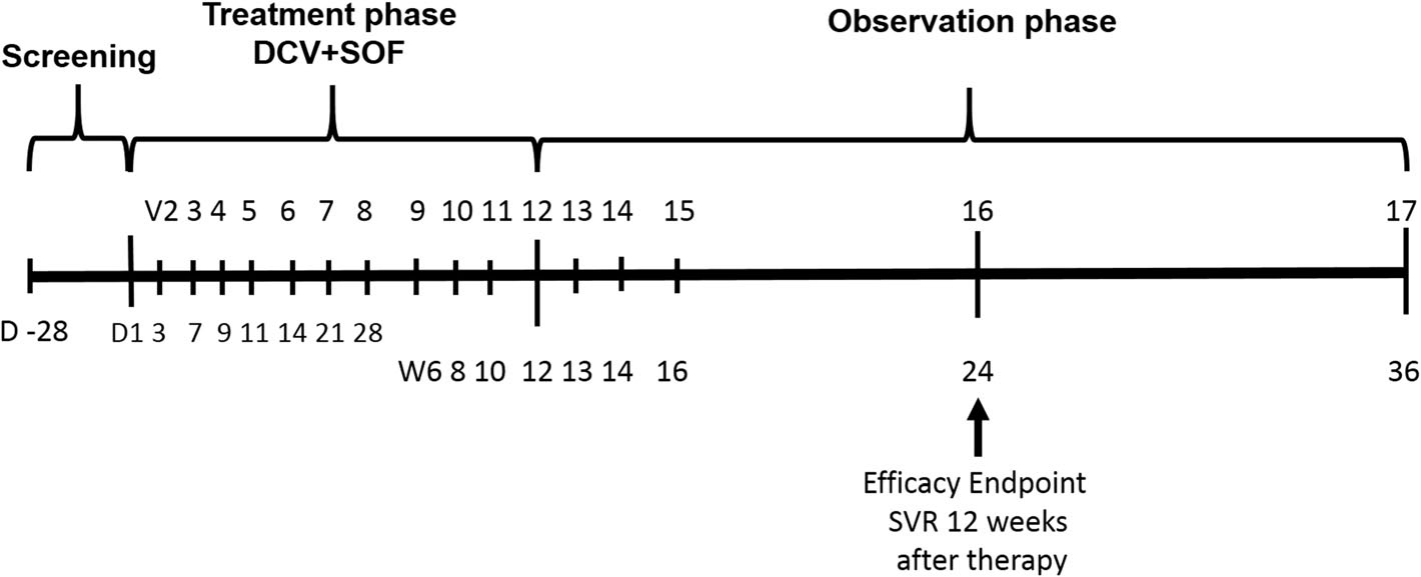
Study design. V = visits, D = days, W = weeks

Secondary efficacy outcomes were the proportion of renal transplant patients with SVR at week 4 (SVR4) and at week 24 (SVR24) after the end of treatment. Viral relapse was defined as confirmed quantifiable or detectable HCV RNA in a study participant with previously HCV RNA unquantifiable or undetectable by nucleic acid testing (NAT) after the end of treatment. In case of viral relapse, combined therapy with DCV (60 mg/d) and SOF (400 mg/d) was extended until week 24. If HCV RNA was still detectable after that extended period, therapy was stopped. Viral breakthrough was defined as confirmed increase in viral load ≥1log value from nadir or any confirmed HCV RNA beyond week 8. In those KTR therapy with treatment was also stopped. At each visit time point HCV-RNA and clinical data were determined to follow closely the response to therapy (see Fig. 1).

#### Safety

We prospectively evaluated safety of treatment regimen at each visit in context of adverse events (AEs), serious AEs (SAEs), suspected unexpected severe adverse reaction (SUSAR) and drug discontinuations. Patient and graft survival including biopsy proven rejections (BPAR) according to BANFF scores [25] were documented. Events of particular interest as infections, clinically significant changes in vital signs or laboratory parameters including new-onset or changes of proteinuria and changes of eGFR at each study visit were captured. In general, participants were encouraged to report any side effects or adverse events at each trial visit.

#### Assessment of glucose metabolism

Consecutive oral glucose tolerance tests (OGTTs) were performed at BL, EOT and SVR12. 2-h plasma glucose concentrations, the preferred WHO diagnostic criteria for KTR, were the primary outcome for diagnosis of post-transplantat diabetes mellitus (PTDM) or impaired glucose tolerance (IGT) [26]. In addition in homeostatic model assessment indexes were assessed to identify insulin resistance and β-cell function (HOMA2-IR/-B) [27].

#### Calcineurin inhibitor assessment

Metabolism rate of tacrolimus (TAC) or ciclosporin A (CyA) treated patients were determined at predefined study visits (Screening, baseline, week (w) 1, 2, 4, 6, 8, 10, EOT, SVR4, − 12 and − 24) by dividing the drug blood trough concentration (C) to the daily TAC or CyA dose (D) respectively.

$$ \mathrm{C}/\mathrm{D}\ \mathrm{ratio}\ \left(\mathrm{ng}/\mathrm{ml}\times 1/\mathrm{mg}\right)=\frac{blood\ (CNI) trough\ level\ \left(\frac{ng}{ml}\right)}{daily\  CNI\  dose\ (mg)} $$

### Staging of liver disease

Percutaneous intercostal ultrasound guided liver biopsies were performed (Acuson-X700, Siemens Erlangen, Germany) with at least three samples of the right lobe using a 18G Quick-Core® Biopsy Needle (William Cook Europe, Bjaeverskov, Denmark) and a 17ga Co-Axial introducer Needle (Argon Medical Devices, Athens, USA). Biopsy samples with a minimum length of 2.0 cm were obtained and directly conserved in 4% Formalin. Because of increased risk of bleeding due to dual thrombocyte anti-coagulation at time of biopsy one patient was biopsied by sheath-mediated (8F) transjugular liver biopsy set (Liver Access and Biopys Needle Set, LABS-200-J, 19G; William Cook Europe). No complications according to Society of Interventional Radiology guidelines were detected in all patients [28].

Histopathological findings were reported and classified according to the modified Scheuer-classification for staging fibrosis and inflammation [29, 30]. Aspartate aminotransferase (AST)-to-platelet ratio index (APRI) and fibrosis-4 score (FIB-4) were calculated as serological markers of fibrosis over the study period [31–33]. In addition, within the scope of screening and clinical observation magnetic-resonance imaging and clinical ultrasound were performed.

### Statistical analysis

For efficacy endpoints all patients were included who received at least one dose of study medication (modified intention to treat population). This is an explorative proof of concept study with a calculated sample size of *n* = 14 patients (power 90% with a type I error of 5% (type II error = 10%) for an estimated efficacy of 79% SVR12. During the course of the study it was decided to enroll 2 additional patients, which even further increases the power of this study to demonstrate adequate efficacy in this population.

Statistical software SPSS version 22 and GraphPad Prism Version 7.0 were used for data analyses.

## Results

### Study population

**Fig. 2:**
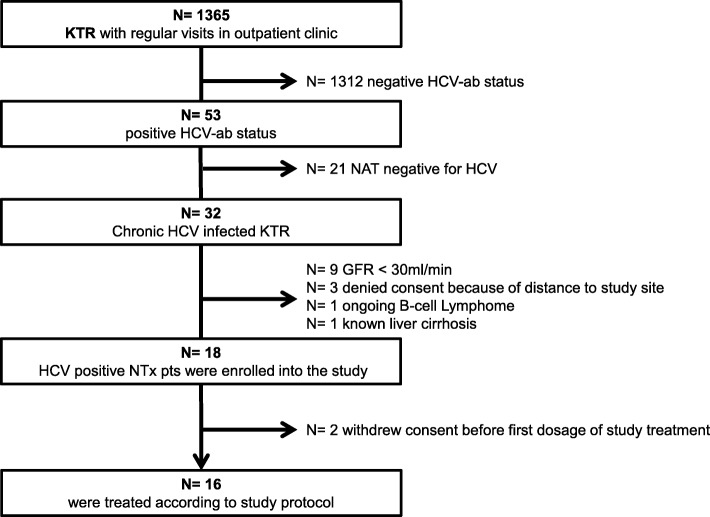
Patient screening strategy to identify study target population

Amongst 1365 KTR that were regularly followed in our outpatient clinic, we identified 32 (2.3%) chronic HCV-infected patients, *n* = 2 with GT1a and *n* = 30 with GT1b respectively. The screening process to identify the target population for this study is summarized in Fig. 2.

Initially, 18 patients met all inclusion criteria and were willing to participate in the study. However, two patients withdrew their consent to participate in the study immediately before any study related procedure was initiated. Therefore, 16 patients received treatment and comprised the study population. Baseline characteristics are shown in Table 1.

**Table 1:** Demographic and Baseline Characteristics

Median Age (range), y	51.5 (34-75)
Sex, male, n (%)	8 (50)
Median time since kidney transplantation (range), y	12.8 (2.3-25.8)
Number of previous renal transplantations (1^st^ /2^nd^/3^rd^/4^th^)	4/11/0/1
Cause of end-stage renal disease, n	
Chronic Glomerulonephritis	5
Polycystic Kidney Disease	3
Alport-Syndrome	4
Interstitial Nephritis	3
Unknown	1
CMV antibody status: (Donor (D+/-)/Recipient (R+/-); n	
Low-risk (D - / R +)	4
Intermediate risk (D + / R +)	10
High-risk (D + / R -)	2
EBV antibody status: (D unknown /R +)	16
Type of Immunsuppressive used, n (%)	
Tacrolimus	8 (50)
Cyclosporine	7 (44)
Sirolimus	1 (6)
Mycophenolate	14 (88)
Azathioprine	1 (6)
Steroids	6 (38)
Renal Transplant function	
Median Creatinine, mg/dl	1.27 (0.95-2.3)
Median eGFR, ml/min per 1.73 m^2^	60 (25-87)
HCV genotype, n	
Ia	1
Ib	15
Median HCV RNA level (range), log_10_ IU/ml	1.19 E^6^ (36600-9.4 E^6^)
Previous HCV therapy (pegINF and/or ribavirin) n/N	4/16
Stage of Liver disease	
Liver biopsy available, n/N	14/16
Stage of Fibrosis, n/N	
• No or minimal (F0, F0-F1, or F1)	2/14
• Moderate (F1-F2 or F2)	10/14
• Severe (F3)	2/14
Median body mass index (range), kg/m^2^	21.47 (16.43-31.25)

### Efficacy and virological response

**Fig. 3:**
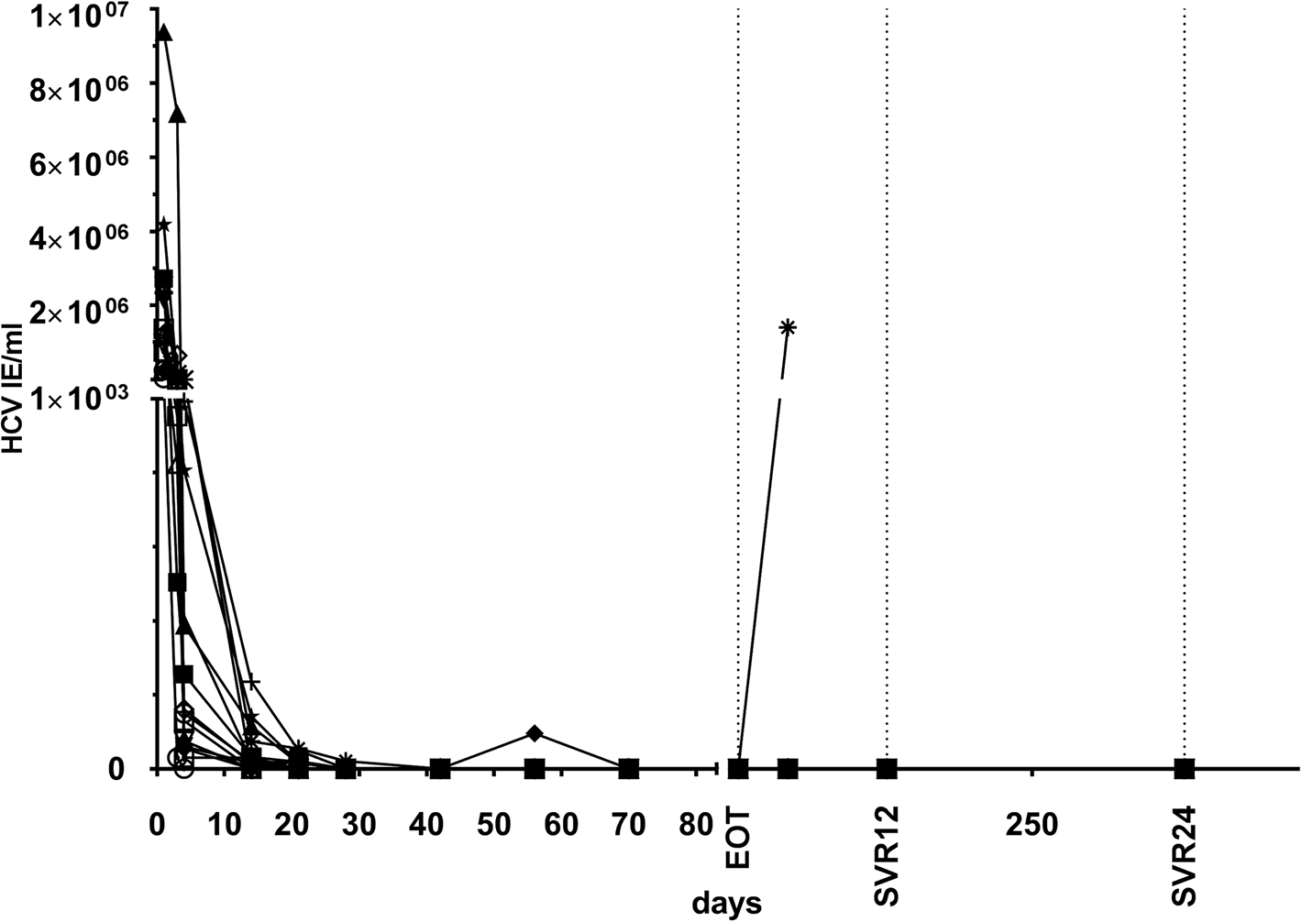
Efficacy of DCV/SOF treatment. HCV-RNA viral load in 16 HCV-positive KTR over the treatment period with DCV/SOF

Mean time to viral clearance was 4 weeks after start of treatment. At EOT, HCV RNA was undetectable in all patients. However, 1 KTR (with genotype 1a) showed viral relapse at week 4 after EOT (time course of viral clearance is shown in Fig. 3). In this patient DCV/SOF, combination therapy was prolonged according to the study protocol for another 12-weeks course. Again, the patient had undetectable HCV RNA at EOT and an early viral relapse 4 weeks later. At this time point ultra-deep sequencing analysis revealed resistance-associated variants (RAVs) for M28-V and Q/R30-R in the NS5A region of the HCV genome in this non-responding patient. In summary, 15 of 16 (94%) KTR achieved SVR4, SVR12 and SVR24 after completion of primary protocol with DCV/SOF.

### Rescue therapy

During the course of this study a new DAA combination therapy containing SOF (400 mg), velpatasvir (VEL, 100 mg), a NS5A inhibitor, and voxilaprevir (VOX, 100 mg), a novel NS3/4A protease inhibitor, was approved for treatment of HCV patients with treatment failure [34]. We initiated this novel therapeutic rescue therapy in the patient with early viral relapse and confirmed RAVs in the NS5A region. After rapid virus decline (HCV RNA not detectable at week 4) HCV RNA has so far remained undetectable in this patient for 12 weeks after EOT.

### Safety data

Overall, DCV/SOF therapy was well tolerated in all 16 KTR with no SUSARs over the study period. In 14 KTR 77 AEs and in 4 KTR 8 SAEs occurred (see Table 2). Five AEs were categorized as suspected to be related to study drug (nausea, reflux and fatigue); no SAE considered as related to the study drug was noticed and no drug discontinuation occurred.

**Table 2:** Safety Lab values

Lab value, (unit)	Baseline, mean (+/-SD)	EOT, mean (+/-SD)	SVR12, mean (+/-SD)	p (one-way ANOVA)
ALT (U/l)	48.31 (31.71)	20.56 (10.52)	19.06 (7.88)	**<0.0001**
AST (U/l)	42.06 (16.78)	27.75 (12.37)	23.0 (12.37)	**<0.0001**
AP (U/l)	87.06 (40.25)	79.69 (40.93)	82.50 (46.18)	0.89
GGT (U/l)	146.13 (282.40)	55.00 (115.15)	53.94 (116.97)	0.29
Bilirubin, total (mg/dl)	0.48 (0.22)	0.31 (0.16)	0.47 (0.29)	0.068
Albumin (g(l)	42.97 (3.48)	43.14 (3.48)	42.67 (4.79)	0.944
Hb (g/dl)	12.9 (1.5)	12.1 (1.35)	12.57 (1.52)	0.31
Ferritin (μg/l)	253.38 (163.37)	124.11 (64.37)	127.33 (63)	**0.002**
HbA1c (%)	5.59 (0.93)	5.33 (0.52)	5.54 (0.95)	0.65
TG (mg/dl)	145.5 (85.58)	147.13 (88.45)	136.06 (91.02)	0.93
HDL (mg/dl)	53.69 (18.54)	55.44 (15.22)	55.81 (15.18)	0.93
CRP (mg/l)	2.44 (2.71)	3.95 (5.82)	3.04 (3.22)	0.59
creatinine (mg/dl)	1.41 (0.44)	1.52 (0.50)	1.54 (0.51)	0.71
proteinuria (mg/g creatinine)	605.63 (1240.54)	374.94 (549.25)	440.94 (689.55)	0.75
HOMA2-IR	89.22 (33.77)	103.3 (36.46)	113.54 (70.35)	0,24
HOMA2-B	128.01 (91.83)	114.21 (63.68)	111.55 (71.23)	0,63

One patient presented with an accidental finding of a post-transplant proliferative lymphoprolferative disease (PTLD) in the initial liver biopsy. At this time point no clinical or laboratory findings were suggestive for an underlying malignancy. In the performed staging CT scan no further extra hepatic PTLD manifestation could be identified. We decided to pursue with DCV/SOF therapy and the patient reached SVR12. After the end of the study patient was treated with four cycles of rituximab (Mabthera® 500 mg). In the follow-up liver biopsy (3 months after end of study) PTLD was no longer detectable.

Regarding graft function, we could not detect any signs of deterioration in renal transplant function, nor any significant changes in proteinuria levels or signs for rejection episodes.

### CNI metabolism

We documented on average an increase of daily dosages of TAC (+ 34.5%) and CyA (+ 21.6%) over the study period. In 4/7 TAC-treated patients an increase of daily dosage was noted. In one patient no changes were necessary and in one patient the dose was decreased after initiation of DCV/SOF. Similarly, in 5/8 CyA-treated patients CyA doses were increased. Again, in one patient CyA dose remained unchanged and in one patient CyA was reduced. In another patient with a sirolimus-based immunosuppressive regimen no adaptation was necessary.

**Fig. 4:**
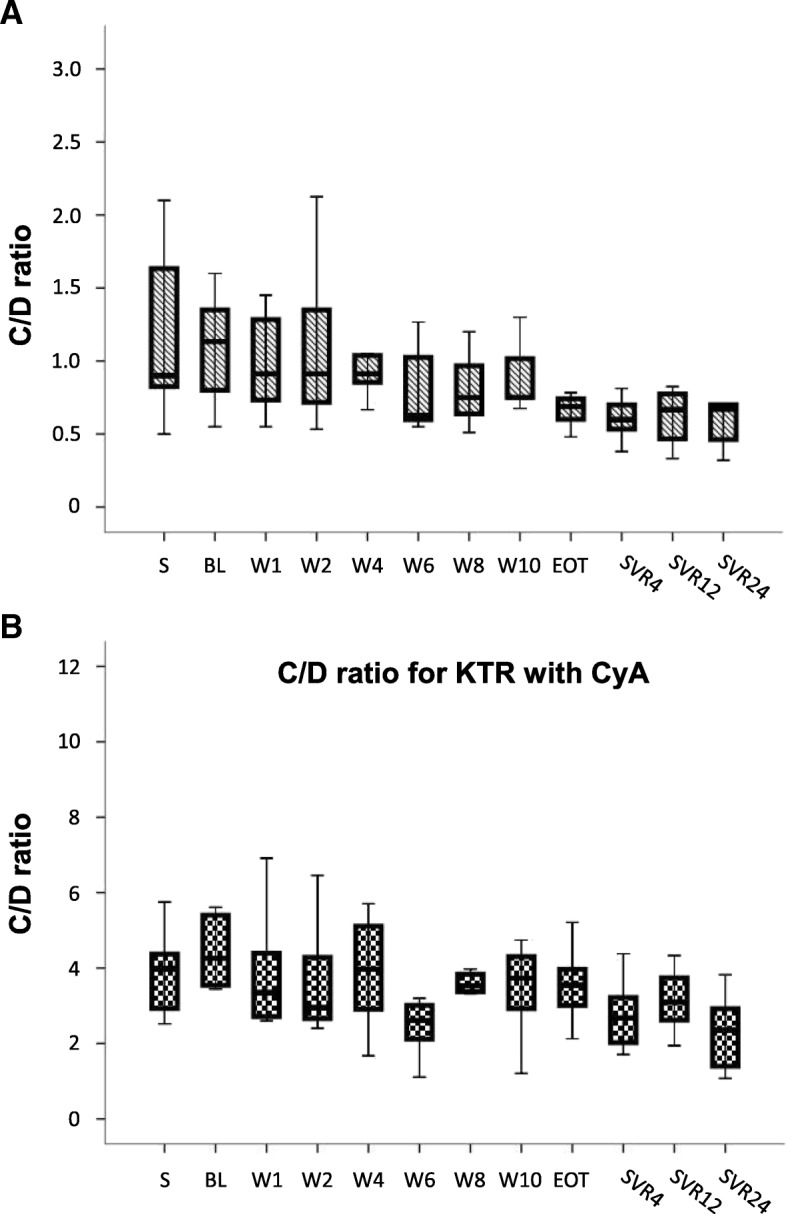
**a** C/D ratio for KTR with TAC. Dose normalized drug trough level concentration (C) to the daily TAC dose (D) is shown. S = screening visit, BL = baseline, W = week, EOT = End of therapy, SVR = Sustanied virological response. **b** C/D ratio for KTR with CyA. Dose normalized drug trough level concentration (C) to the daily CyA dose (D) is shown. S = screening visit, BL = baseline, W = week, EOT = End of therapy, SVR = Sustanied virological response

To further specify the drug metabolism we assessed the C/D ratio of TAC-(*n* = 7) and CyA-(*n* = 8) treated patients. After initiation of therapy TAC C/D ratio declined over the study period (BL: 4.33+/− 1.5; EOT: 2.85+/− 0.84; SVR12: 2.49+/− 0.76; SVR24: 3.12+/− 2.19; (mean +/-SD; *p* = 0.003, Friedman test, see Fig. 4a).

In CyA treated patients the course of C/D scores was similar: BL: 1.31+/− 0.71; EOT: 0.89+/− 0.23; SVR12: 0.79+/− 0.21; SVR24: 0.57+/− 0.23; (mean +/-SD; *p* = 0.0001, Friedman test, see Fig. 4b). Thus, therapy with DCV/SOF apparently increased the metabolism of both CNIs. Reduced C/D scores were documented for both CNIs until the end of study (week 36/SVR24).

### Hepatic function

In 14 of 16 patients a liver biopsy was performed at BL (two patients dropped out; one of them due to advanced polycystic liver disease, one withdrew consent). Histopathology in the 14 patients showed a median fibrosis score of 2 (shown in Table 1), a median inflammation score of 1 and a medium fat deposition of 5% (modified Scheuer-classification).

Changes of laboratory values are displayed in Table 3. Liver parameters (ALT and AST) as well as ferritin levels improved significantly after HCV treatment (*p* < 0.0001). APRI (0.47+/− 0.22 vs. 0.25+/− 0.11; (mean +/-SD); *p* = 0.001) and FIB-4 score (1.45+/− 0.63 vs. 1.19+/− 0.53; (mean +/-SD); *p* = 0.023) also improved significantly over the study period. There were no significant changes in conventional MRI or ultrasound imaging during the study (data not shown).

**Table 3:** Safety Analysis and overall Adverse Events

	**DCV + SOF (n=16)**			
**Parameter**	*until EOT*	*until end of study*		
No. of all AEs	43	77		
AEs suspected of being related to study drug	5	5		
No. of SAEs	2	8		
No. of SAEs which were in relation to study drug	0	0		
No. of patients with one or more AE	12	14		
No. of patients with one or more SAE	2	4		
No. of patients who needed hospitalization	2	7		
			**AE Drug related**	**SAE**
Gastrointestinal events, no., (% of al AEs)	5 (11.63%)	6 (7.79%)		
Nausea	3	3	yes	
Diarrhea	1	1		
Gastro-enteritis	2	2		
Reflux/Gastritis	1	2	yes	
Infection related events, no. (% of all AEs)	17 (39.53%)	31 (40.26%)	no	
Urinary tract infection	5	9		2
Sepsis, 3-MRGN	0	1		1
C. difficile infection	1	1		1
Common cold	4	10		
Pneumonia/Bronchitis	2	3		1
Fever	1	2		
Conjunctivitis	0	1		
Epiglottitis	1	1		
Tonsillar angina	1	1		
No. of any Efficacy failure (%) of patients	0 (0%)	1 (6.25%)		
Viral breakthrough	0	0		
Viral relapse	0	1		1
Any Malignancies or any suspected Neoplasia , no.(%) of patients^#^	1 (6.25%)	2 (12.5%)		
PTLD* in liver	1	1		
Follow-up liver biopsy	0	1		1
Pituitary adenoma	0	1		1
Any Heart, Cardiovascular, no. (%) of all AEs	2 (4.65%)	2 (2.6%)		
Tachycardia	1	1		
Hypertension	1	1		
Any Neurological events, no. (%) of all AEs	4 (9.3%)	9 (11.69%)		
Concentration weakness	1	2		
Headache	3	4		
Dizziness	0	1		
Hyposphagma	0	1		
No. of any other AE. (%) of patients	14 (32.56%)	26 (33.77%)		
Asthma episode/ Dyspnoe	1	5		
Myalgia	2	4		
Lower back pain	2	3		
Fatigue	1	2	yes	
Anemia/Iron deficiency	3	3		
Alopecia	1	1		
Leukocytosis	1	1		
Urinary incontinence	1	1		
Hyperkaliemia	0	1		
Hypercalcemia	0	1		
Edema	2	2		
Pancreatic cyst	0	1		
Rib fracture	0	1		

### Glucose tolerance

One patient was excluded from the analysis because of preexisting diabetes mellitus type I. Initial performed OGTT at baseline identified one patient with PTDM (2 h-gluc ≥200 mg/dl) and four patients with IGT (2 h-gluc ≥140 mg/dl), whereas all the other 10 patients had OGTTs within normal limits. Further evaluations at SVR12 provided pre diabetic values in scheduled OGTTs in two out of 14 patients only. One patient did not consent to the SVR12 OGTT. ANOVA demonstrated OGTT-derived 2 h-glucose differed significantly between scheduled visits (Wilks-Lamdba =0.405, F (2, 11)=8.081, *p* = 0.007). At BL mean 2 h-glucose was 141 ± 77 mg/dl, 132 ± 52 mg/dl at EOT and 117 ± 77 mg/dl at SVR12. Finally, between BL and SVR12 paired samples t-test confirmed significant differences in 2 h-glucose.

We did not find any statistical significant changes in HOMA2-IR/-B (Table 3), but results directed to improved insulin sensitivity and decreased beta cell output correspondingly. This reflects consistency with overall improved glucose tolerance.

## Discussion

Here we present the first prospective, open-label study with a DCV/SOF based DAA regimen in chronically HCV-infected KTR. Our data shows that the use of this pan-genotypic combination therapy is safe and effectively cures chronic and long-standing HCV disease in KTR with GFR > 30 ml/min/1.73m^2^ and no signs of advanced liver disease.

In total, 15/16 KTR (94%) achieved SVR12 with DCV/SOF combination. In these “responders”, we noticed early viral response, defined as a rapid virus clearance already after a median of 4 weeks after initiating DCV/SOF therapy. One patient achieved negative HCV PCR at EOT but had early viral relapse 4 weeks after EOT. Based on the protocol a second 12-weeks therapy extension with DCV/SOF was performed, but the patient developed another early viral relapse. Deep sequencing HCV genome analysis revealed RAVs to all available viral NS5A inhibitors [35]. Therefore, we administered a 12-weeks course of SOF/VEL/VOX (Vosevi®). This fix dose regimen was approved by the FDA on July 18th, 2017 for patients with any genotype of chronic HCV infection, without cirrhosis or with compensated cirrhosis and previously failed therapy with a DAAs-containing regimen [34]. Here we report the first successful treatment with this novel combination therapy as a rescue therapy in a KTR with chronic HCV infection and detected NS5A-RAVs.

Over the entire study period we reported 77 AEs, 8 were categorized as SAE. Until EOT, 43 AEs and 2 SAEs were documented. However, in only three study subjects mild AEs were categorized as drug-related, which reflect excellent tolerability of DCV/SOF. In no case the drug regimen was discontinued. In addition, we noticed stable graft function without any evidence for BPARs or worsening of proteinuria over the study period. Our findings are in contrast to Lubetzky et al. who most recently reported a retrospective analysis of 31 KTR with worsening of pre-existing proteinuria and a decline of renal function under DAAs therapy [36].

Because blood levels of CyA or TAC may be affected by drug-drug interactions, dosages and trough levels were analyzed prospectively. As per center practice, the maintenance immunosuppressive regimen of all patients was continued during this study. We found significant changes in the TAC and CyA C/D ratios in the blood after initiation of DCV/SOF. Due to lower trough levels of immunosuppressants daily dosage of TAC and CyA in most patients were increased over the study course. Dose normalized ratios for TAC and CyA were further decreasing after EOT until the end of study. These findings are in line with other published cohorts [22, 37]. Of note, neither SOF nor DCV interacts directly with cytochrome P450, in fact this pan-genotypic combination therapy seems very favorable in terms of drug-drug interactions in KTR [38]. We rather speculate that improvement of hepatic function increased the ability of CYP3A4 to metabolise TAC and CYA, resulting in higher dose requirements to reach target levels in study participants.

At study entry, most of the patients showed moderate signs of liver fibrosis and hepatic inflammation in the liver biopsy. In MRI and ultrasound no signs for liver cirrhosis were detectable in our population. During the study we found a significant improvement of liver function displayed by a significant reduction of initially elevated liver enzymes and improvement of APRI and FIB-4 scores. However, ultrasound and MRI showed no significant differences over the study period.

Patients with chronic HCV infection also frequently show serum and hepatic iron overload due to lower hepcidin levels [39]. During our study we found a significant reduction of ferritin levels after successful cure of HCV infection, which might be caused by improved hepatic function after HCV treatment.

One patient showed incidental asymptomatic HCV associated B-cell lymphoma in the initial biopsy finding. We decided to proceed with the planned study protocol as a potential causative treatment option. The patient received rituximab as a specific PTLD treatment after the end of study. Follow-up MRI and liver biopsy, which was performed 3 months after end of study, showed no signs of PTLD. Although it is well established that regression of HCV associated lymphatic disorders is closely correlated with viral clearance at least with interferon-based antiviral therapy, data on resolution of PTLD after treatment with DAAs are very limited so far [40, 41].

HCV infection is known to induce metabolic changes, such as insulin resistance and beta-cell dysfunction [42]. Virus clearance improves insulin resistance, β-cell function, and hepatic expression of insulin receptor substrate 1 and 2 [43]. Clinically relevant improvements in glucose tolerance have been shown in the non-transplant population by OGTT [44]. KTR are particularily vulnerable to develop PTDM when HCV infection coincides.

OGTT is the preferred method to assess carbohydrate tolerance in stable KTR and allows for diagnosis of IGT, an independent risk factor of subsequent PTDM [26]. Within this trial, KTR previously in the pre-diabetic range returned to normal glucose tolerance. Among all patients, 2-h glucose improved significantly. In addition, we noticed a trend for improvement in HOMA2-IR/-B scores. As PTDM is the principal determinant of death with functioning graft after kidney transplantation, this antidiabetic effect could possibly translate into improvement of overall outcomes.

Our study has some limitations. Although currently the largest prospective trial in KTR with DCV/SOF, the number of 16 treated patients is still relatively small. In addition, further follow-up will have to proof sustained viral clearance and functional improvement. Given the nature of the intervention, the small size of the study population and the availability of objective response parameters, we decided to implement an un-controlled open-label design. The study population had mostly GT1b HCV infection and the relevance for other genotypes has to be determined. The strength of the study includes the prospective design, the systematic assessment of AEs and the measurement of both, viral response and functional parameters.

## Conclusions

Our study shows safety and efficacy of DAAs in KTR. Based on the observed functional improvements there appears to be potential benefit for clinical outcomes. However, mostly because of improvement of liver function careful surveillance of immunosuppressive trough levels appears mandatory, as the necessity for dose adjustments is common. We recommend testing for RAVs prior to start of a specific DAAs regimen. As “second-line” DAAs for patients with previously failed DAAs therapy are nowadays available, there are also treatment options for non-responders and relapsers. Moreover, the acceptance of HCV positive donor organs for HCV negative transplant candidates is an option that deserves further studies [45].

## Abbreviations

AEs: Adverse events
APRI: AST to-platelet ratio index
AST: Aspartate aminotransferase
BPAR: Biopsy proven rejections
CyA: Ciclosporin A
DAA: Direct acting antiviral
DCV: Daclatasvir
EOT: End of treatment
FIB-4: Fibrosis-4 score
HCV: Hepatitis C Virus
HOMA2-IR/-B: Homeostatic model assessment indexes were assessed to identify insulin resistance and β-cell function
IGT: Impaired glucose tolerance
KTR: Kidney transplant recipient
NAT: Nucleic acid testing
OGTT: Oral glucose tolerance test
pegIFN: Pegylated interferon
PTDM: Post-transplantat diabetes mellitus
PTLD: Post-transplant lyphoproliferative disease
RBV: Ribavirin
SAEs: Serious AEs
SOF: Sofosbuvir
SUSAR: Suspected unexpected severe adverse reaction
SVR: Sustained virological response
TAC: Tacrolimus
ULN: Upper limit of normal
VEL: Velpatasvir
VOX: Voxilaprevir
